# Circ_0136474 and MMP‐13 suppressed cell proliferation by competitive binding to miR‐127‐5p in osteoarthritis

**DOI:** 10.1111/jcmm.14400

**Published:** 2019-08-11

**Authors:** Zhao Li, Bo Yuan, Zheng Pei, Keshi Zhang, Zhentao Ding, Si Zhu, Yichuan Wang, Zhenpeng Guan, Yongping Cao

**Affiliations:** ^1^ Center of Arthritis Clinic & Research Peking University People’s Hospital Beijing China; ^2^ Department of Orthopedics Peking University Shougang Hospital Beijing China; ^3^ Department of Orthopedics Civil Aviation General Hospital Beijing China; ^4^ Department of Orthopedics Peking University First Hospital Beijing China

**Keywords:** Circ_0136474, miR‐127‐5p, MMP‐13, Osteoarthritis

## Abstract

Osteoarthritis (OA) is a prevalent degenerative joint disease whose pathogenesis remains unclear. The research aims to investigate the roles of Circ_0136474/miR‐127‐5p/MMP‐13 axis in OA. Differentially expressed circRNAs and miRNAs in OA cartilage tissue were screened out and visualized by R project based on RNA‐seq data and microarray data respectively. qRT‐PCR was carried out for detection of relative expression levels of Circ_0136474, miR‐127‐5p, MMP‐13 and other inflammatory factors and Western blot analysis was conducted to detect the protein expression level of MMP‐13. CCK‐8 assay and flow cytometry were conducted to determine cell proliferation and cell apoptotic ability respectively. RNA‐fluorescence in situ hybridization (RNA‐FISH) experiments were conducted to confirm the immune‐localization of the Circ_0136474 and MMP‐13 in human tissues. Targeted relationships were predicted by bioinformatic analysis and verified by dual‐luciferase reporter assay. Our findings revealed that the expression levels of both Circ_0136474 and MMP‐13 in OA cartilage tissue were significantly higher than that in normal cartilage tissue. Circ_0136474 could suppress cell proliferation by facilitating MMP‐13 expression and suppressing miR‐127‐5p expression in OA. Overexpression of miR‐127‐5p negatively regulated MMP‐13 expression to enhance cell proliferation. Our study demonstrated that Circ_0136474 and MMP‐13 suppressed cell proliferation, while enhanced cell apoptosis by competitive binding to miR‐127‐5p in OA, which may well provide us with a new therapeutic strategy for osteoarthritis.

## 1 INTRODUCTION

Osteoarthritis (OA) is one of the most common joint diseases mainly characterized by the degradation of the articular cartilage and osteophyte formation,^1^, ^2^ which is also regarded as the most common chronic and potentially irreversible disease that affects the joints globally.^3^ OA is highly prevalent among the elder and also one of the three most frequent causes of disability in younger people.^4^ Although OA therapy has been significantly upgraded, more optimal and integrated treatment has yet to be developed.^5^ Hence, it is vital to study underlying mechanisms of OA pathogenesis, which will provide us with new therapeutic strategies for treatment of OA.

Circular RNAs (circRNAs) are endogenous RNAs featuring for covalent loop structures with neither 5′ to 3′ polarity nor a polyadenylated tail.^6^ It was reported that circRNAs regulate the gene expression in both transcriptional and post‐transcriptional process.^7^ Recently, circRNAs, novel noncoding RNAs with varied biological functions and pathological implications, have been shown to be closely related with various diseases, including osteoarthritis, atherosclerotic vascular disease risk, neurological disorders, Parkinson's disease, Alzheimer's disease, schizophrenia and so on.^8^ However, the roles of circRNAs, as well as circRNA and miRNA axis in the development and progression of OA are little understood. In this study, our attention is mainly fixed on the function of Circ_0136474 in OA progression.

miRNA is one of the most eminent classes of ncRNAs and involved in several aspects of gene regulation in eukaryotes. miRNAs always serve as fine‐tuning regulators such as onco‐miRNAs or tumour‐suppressor miRNAs for diverse biological processes, which is mainly dependent on its downstream genes.^9^ Previous studies showed that miR‐9 was up‐regulated in late‐stage OA cartilage and directly targeted MMP‐13.^10^ Besides, Okuhara et al reported that miR‐146a could also directly target MMP‐13 and was significantly higher during the early stages of OA than during the later stages.^11^ Based on latest research, miR‐127‐5p regulated MMP‐13 expression and IL‐1β‐induced catabolic response in human chondrocytes.^12^

Matrix metalloproteinases (MMPs) family function in irreversible matrix degradation and subsequent joint destruction in OA.^13^ Previous reports showed that Interleukin‐1β (IL‐1β) and tumour necrosis factor‐α (TNF‐α) are two most potent catabolic factors that increase the production of inflammatory mediators and the expression of MMPs in chondrocytes.^14^ Among these MMPs, MMP‐13 has caught a lot of attention in that it was significantly overexpressed in both joints and articular cartilage in OA patients and could hardly be detected in normal tissues. Besides, MMP‐13 was known to function as an extracellular matrix (ECM)‐degrading enzyme in OA joints.^15^, ^16^ In clinical samples, MMP‐13 was abnormally expressed during different stages of OA process and was found to up‐regulated during the early stage and down‐regulated during the late stage in human OA cartilage, which indicated its central node in the cartilage degradation network and contribution of MMP‐13 to the initiation/onset of OA.^17^, ^18^ In addition, the activity of MMP‐13 can be regulated at multiple levels, including epigenetic modification, transcriptional regulation and post‐transcriptional regulation by ncRNAs.^19^, ^20^ Furthermore, it was revealed that MMP‐3 and MMP‐13 were both up‐regulated in articular chondrocytes of OA patients.^21^

Along with the illumination of circRNAs acting as endogenous miRNA sponges by competitive binding to miRNA response elements to suppress their expression, a new mode of action for circRNAs was unveiled.^22^ Interaction between miRNA and circRNAs has already been validated to be very significant in cases of osteoarthritis, gastric cancer, colorectal cancer and neurodegenerative pathologies. For instance, circRNA‐CER (Circ_100876) serves as a miR‐136 sponge, sequestering miRNAs and controlling the expression level of MMP13 and participating in the process of chondrocyte ECM degradation in OA.^23^

Although quite a lot of miRNAs have been reported to play a pivotal role in OA,^24^ the specific micro‐environmental factors that are involved in this pathological process have not been fully elucidated. Therefore, researches on the effects of both miRNAs and their potential circRNA regulators on OA progression may help clarify the pathogenesis of OA.

Our study showed Circ_0136474 promoted MMP‐13 expression via inhibiting miR‐127‐5p, which might well provide us with more potential and therapeutic strategies for treatment of OA.

## 2 MATERIALS AND METHODS

### 2.1 High‐throughput sequencing analysis

High‐throughput sequencing data of 18 normal tissue samples and 20 OA human knee cartilage tissue samples were obtained from Gene Expression Omnibus (GEO) (https://www.ncbi.nlm.nih.gov/geo/query/). The series accession number was GSE114007 and corresponding platform was GPL11154. Given that the sequencing quality of one normal knee cartilage tissue sample was not as good as we expected, only 17 normal tissue samples and 20 OA human knee cartilage tissue samples were employed for differential analysis. The expression values of circRNAs in normal cartilage tissues and OA cartilage tissues were obtained after quantification and background correction. Differentially expressed circRNAs were screened out with the screening criteria |log_2_ (Fold Change)|>1 and adjusted *P* value < 0.05 by limma package. Top 20 differentially expressed circRNAs (7 up‐regulated and 13 down‐regulated) were visualized by heatmap.

### 2.2 Microarray analysis

Microarray data of miRNA expression profile in OA were downloaded from GEO (https://www.ncbi.nlm.nih.gov/geo/query/). The series accession number was GSE105027 and matched platform was GPL21575. In brief, data were filtered out and normalized for selecting differentially expressed miRNAs with selecting criteria adjusted *P* value < 0.05 and |FC (Fold Change)|>2 by limma package. Besides, miRNAs which potentially targeted Circ_0136474 were predicted by c*ircular RNA Interactome* (https://circinteractome.nia.nih.gov/) and listed in Table S1. Based on c*ircular RNA Interactome* (https://circinteractome.nia.nih.gov/), miR‐127‐5p was predicted to target Circ_0136474. Besides, OA‐related miRNAs, including miR‐127‐5p were singled out and listed in Table S2 based on HMDD (http://www.cuilab.cn/hmdd). Finally, miR‐127‐5p showed up in three tables mentioned above and Circ_0136474/miR‐127‐5p were selected for further investigation.

### 2.3 Clinical samples

A total of seven OA cartilage samples were obtained from OA patients who underwent total knee arthroplasty. All of OA patients were pathologically and cytologically diagnosed according to American College of Rheumatology criteria. Normal articular cartilage was obtained from seven trauma patients with no history of OA. Informed consent was signed by all tissue donors involved in this study. The research was allowed by the Ethics Committee of Peking University First Hospital.

### 2.4 RNA‐FISH assay

The FISH assay was performed according to the manufacturer's protocol (Sino Biological Inc, Beijing, China). Briefly, after the fixed slides being dehydrated, the probes specific to Circ_0136474 and MMP‐13 were added to the slides and then pre‐denatured at 78℃ for 5 minutes. Hybridization was then carried out at 42℃ overnight. Nuclei were counterstained with 4,6‐diamidino‐2‐phenylindole (DAPI, Sigma). Images were examined with a Zeiss LSM 700 Meta confocal microscope (Jena, Germany).

### 2.5 Cell culture and cell transfection

Human OA articular chondrocytes were isolated from articular cartilage of OA knee joints. In brief, articular cartilage was dissected and subjected to sequential digestion with both collagenase B and pronase in DMEM (Thermo Fisher Scientific, China). Thereafter, isolated articular chondrocytes were centrifuged and washed several times with PBS. OA articular chondrocytes were maintained in DMEM supplemented with 10% foetal bovine serum (FBS; Thermo Fisher Scientific, China), 100 U/mL penicillin and 100 μg/mL streptomycin (Gibco‐BRL, Gaithersburg, MD, USA) at 37℃ in a humidified atmosphere 5% CO_2_. OA articular chondrocytes needed to be passaged twice or three times for use in subsequent experiments. Cell transfections were all performed with Lipofectamine 2000 reagent (Invitrogen, Carlsbad, CA, USA) following the manufacturer's protocols. si‐Circ_0136474‐1 and si‐Circ_0136474‐2 (small interfering RNAs for Circ_0136474), pcDNA3.1‐Circ_0136474, si‐MMP‐13‐1 and si‐MMP‐13‐2, miR‐127‐5p mimics, miR‐127‐5p inhibitor and negative control (NC) were all purchased from GenePharma (China).

### 2.6 qRT‐PCR

TRIzol^TM^ Reagent (Thermo Fisher Scientific) was utilized for isolation of total RNA. RNase‐R digestion treatment was conducted at 37°C by using RNase‐R (Epicenter) 2 U/mg for 25 minutes. Thereafter, RNA was reversely transcribed into cDNA with Reverse Transcription Kit (Takara, Dalian, China). Subsequently, qRT‐PCR was carried out by SuperReal SYBR Green kit (Thermo Fisher Scientific) following manufacturer's instructions. The expression levels of mRNAs were normalized against GAPDH and relatively quantified using 2^−ΔΔCt^ method. The expression levels of miRNA and circRNA were normalized against U6 and relatively quantified using 2^−ΔΔCt^ method. The primers for qRT‐PCR in this study are enlisted in Table 1.

**Table 1:** Primers used in this study

Gene	Sequence
Circ_0136474‐F	TGCCATCACAGTGGGAATAC
Circ_0136474‐R	GGTTGTCATAAGCAGGACCA
MMP‐13‐F	GCAGTCTTTCTTCGGCTTAGAG
MMP‐13‐R	GTATTCACCCACATCAGGAACC
TNF‐α‐F	GCCAGAGGGCTGATTAGAGA
TNF‐α‐R	TCAGCCTCTTCTCCTTCCTG
IL‐17‐F	CCACCTCACCTTGGAATCTC
IL‐17‐R	CAGGATCTCTTGCTGGATGG
IL‐1β‐F	CGATCGCGCAGGGGCTGGGCGG
IL‐1β‐R	AGGAACTGACGGTACTGATGGA
GAPDH‐F	GGAAGGTGAAGGTCGGAGTC
GAPDH‐R	CGTTCTCAGCCTTGACGGT
U6‐F	TGCGGGTGCTCGCTTCGGCAGC
U6‐F	CCAGTGCAGGGTCCGAGGT
Collagen II‐F	CAAGTCGCTG AACAACCAGA
Collagen II‐R	GCCCTCATCTCCACATC ATT

Note

F, Forward primer; R, Reverse primer.

### 2.7 Dual‐luciferase reporter gene assay

The pmirGLO luciferase reporter gene vector (Promega, Madison, WI, USA) with either Circ_0136474 WT or Circ_0136474 MUT was co‐transfected with miR127‐5p mimics or mimics control into HEK‐293 cells using Lipofectamine 2000 reagent (Invitrogen). Similarly, the pmirGLO luciferase reporter gene vector (Promega, Madison, WI, USA) loaded with either MMP‐13 WT or MMP‐13 MUT was co‐transfected with miR127‐5p mimics or mimics control into HEK‐293 cells using Lipofectamine 2000 reagent (Invitrogen). The relative luciferase activities should be measured 48 hours after transfection.

### 2.8 Western Blot

Total proteins were isolated by RIPA buffer (Solarbio, China) and quantified by BCA Protein Assay Kit (Beyotime, Shanghai, China). Samples ran SDS‐PAGE for about 2.5 hours, then were transferred to the polyvinylidene fluoride (PVDF) membranes (Invitrogen). The membranes were then blocked by 3% Bovine Serum Albumin (BSA) (Sigma‐Aldrich). Afterwards, primary antibodies were added as well as incubated overnight at 4℃. Next, secondary antibody horseradish peroxidase (HRP)‐conjugated goat‐anti‐rabbit Immunoglobulin G (IgG) (ab205718, 1:2000, Abcam) was added and incubated at 37℃ for 1 hours. Primary antibodies were as follows: rabbit anti‐MMP‐13 (ab38012, 1:5000, Abcam) and rabbit anti‐GAPDH (ab9485, 1:2500, Abcam). Primary antibodies were all purchased from Abcam. Images were obtained by a Tanon‐4200 Chemiluminescent Imaging System (Tanon, China).

### 2.9 Cell Counting Kit‐8 (CCK‐8) assay

Cells were seeded in 96‐well plates at the density of 2 × 10^4^ cells/mL and then incubated at 37°C. CCK‐8 reagent (Beyotime Institute of Biotechnology, Haimen, China) was added into each well of a 96‐well plate for 30 minutes at 37°C and the OD values were read at 490 nm.

### 2.10 Flow cytometry assay

Cell apoptotic rate was detected following the manufacturer's protocols of Annexin V‐FITC/PI Apoptosis Detection Kit (KeyGEN Biotech, Nanjing, China). After successively staining with both Annexin V‐FITC and PI for half an hour, cells were incubated in a dark room for 25 minutes. Afterwards, the status of cell apoptosis was detected by the MoFlo flow cytometer (Beckman Coulter, Atlanta, GA, USA).

### 2.11 Statistical analysis

ANOVA, along with the *t* test were adopted to test the statistical differences among multiple groups. Data were obtained from at least three independent tests. All statistical analysis was carried out using GraphPad Prism 6.0 software (La Jolla, CA, USA). The differences were deemed statistically significant with * *P* < 0.05.

## 3 RESULTS

### 3.1 Circ_0136474 was up‐regulated in OA cartilage tissues and could be targeted by miR‐127‐5p

**Figure 1:**
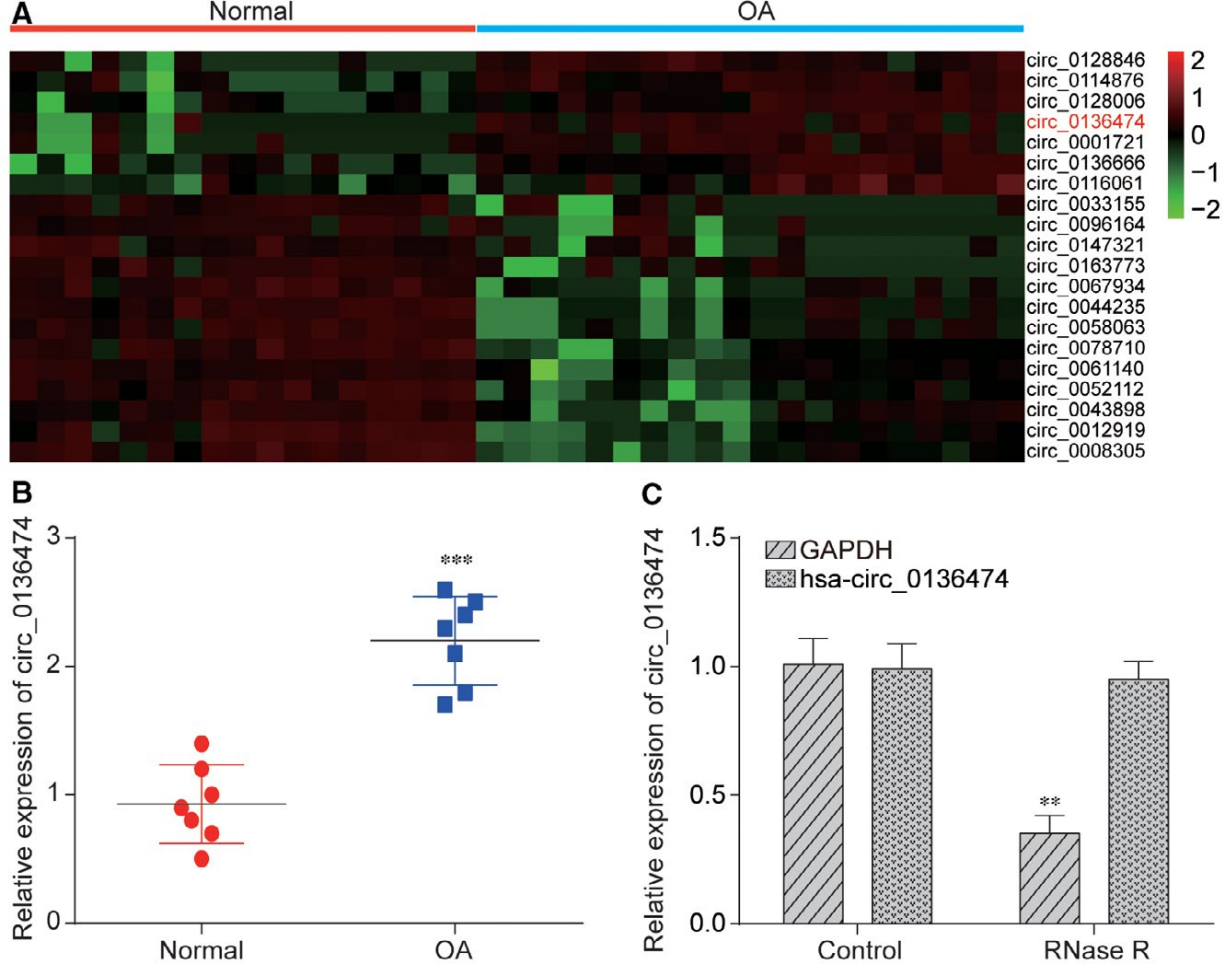
Circ_0136474 expression was significantly higher in OA than in normal tissues. A, Heatmap contains expression profiles of 20 different circRNAs between normal tissues and OA cartilage tissues and Circ_0136474 showed a robust increase in OA. B, It was verified by qRT‐PCR that Circ_0136474 was significantly higher in OA cartilage tissues than in normal cartilage tissues. C, Linear RNA can be digested by RNase R and GAPDH expression was significantly decreased after digested with RNase R while Circ_0136474 has not been affected significantly. **P < 0.01 and ***P < 0.001 meant that there was a significant difference compared with NC group

Differentially expressed circRNAs between OA cartilage tissues and normal cartilage tissues were filtered out, top 20 of which were visualized in heat map in Figure 1A. Among them, Circ_0136474 showed a robust increase in OA. And we further detected the expression level of Circ_0136474 in OA tissues and normal tissues. Figure 1B revealed that relative expression of Circ_0136474 was largely higher in OA than in normal tissues. In general, RNase R can digest liner RNA but not circRNA. As shown in Figure 1C, the relative expression of GAPDH was greatly decreased after digestion by RNase R and the relative expression of Circ_0136474 was not affected, suggesting the cyclic structure of Circ_0136474.

**Figure 2:**
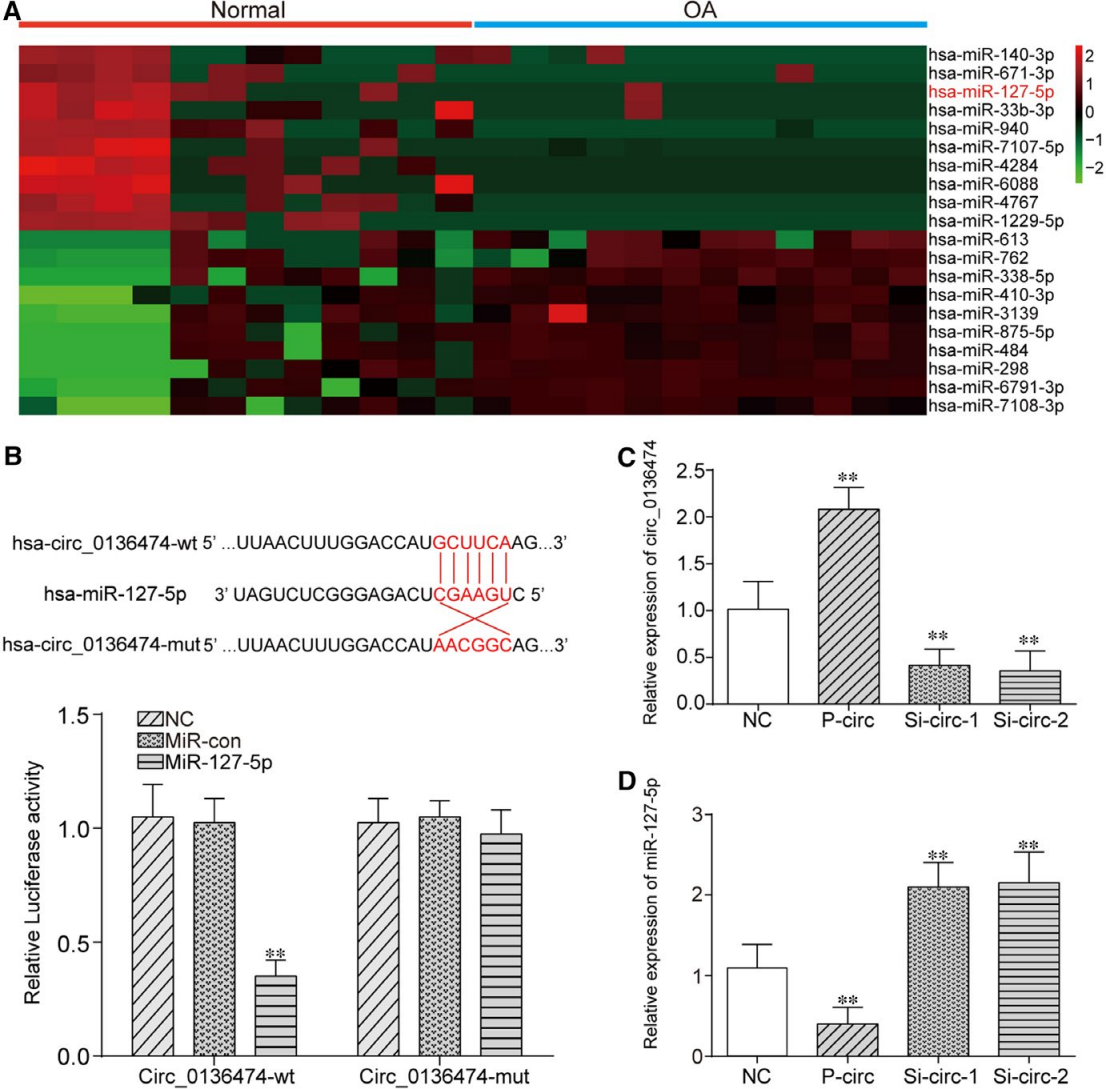
Circ_0136474 could bind to the miR‐127‐5p and suppress its expression. A, Visualization of 20 different miRNAs between normal cartilage tissues and OA cartilage tissues and miR‐127‐5p was significantly down‐regulated in OA. B, The predicted targeted relationship between Circ_0136474 and miR‐127‐5p using *Circular RNA Interactome* (https://circinteractome.nia.nih.gov/ ). Dual‐luciferase reporter gene assay verified the targeted relationship between Circ_0136474 and miR‐127‐5p. C, p‐circ could greatly up‐regulate the expression of Circ_0136474, which could be instead suppressed with the silencing of Circ_0136474. D, Overexpression of Circ_0136474 could greatly suppress the expression of miR‐127‐5p, which could otherwise be reversed with the knockdown of Circ_0136474. **P < 0.01 meant that there was a significant difference compared with NC group

Furthermore, differentially expressed miRNAs between OA cartilage tissues and normal tissues were filtered out from GSE105027 and visualized in heat map in Figure 2A. Among them, miR‐127‐5p was predicted to target Circ_0136474 based on c*ircular RNA Interactome* and closely related with OA progression based on HMDD. Therefore, Circ_0136474/miR‐127‐5p was selected for further investigation in this study. After further analysis of the sequence of Circ_0136474, targeted relationship was found between miR‐127‐5p seed region and Circ_0136474 3’UTR region based on c*ircular RNA Interactome*. Relative luciferase activity in that group co‐transfected with miR‐127‐5p mimics and Circ_0136474‐WT was lower than that of group with co‐transfection of both miR‐127‐5p NC and Circ_0136474‐WT, revealing the direct binding between miR‐127‐5p and Circ_0136474 in OA (Figure 2B, *P* < 0.01). Subsequently, qRT‐PCR was employed for validation of transfection efficiency. Figure 2C showed us that overexpression of Circ_0136474 tremendously increased Circ_0136474 expression, which was instead largely reduced with either si‐Circ_0136474‐1 or si‐Circ_0136474‐2 (*P* < 0.01). Given that knockdown efficiency of si‐Circ_0136474‐2 was better than si‐Circ_0136474‐1, si‐Circ_0136474‐2 was selected for further experiments. Besides, Figure 2D revealed that overexpression of Circ_0136474 largely decreased miR‐127‐5p expression, which was otherwise increased with si‐Circ_0136474‐1 or si‐Circ_0136474‐2 (*P* < 0.01). In brief, Circ_0136474 was up‐regulated in OA cartilage tissues and could be targeted by miR‐127‐5p.

### 3.2 miR‐127‐5p could directly target MMP‐13 and suppress its expression in OA

**Figure 3:**
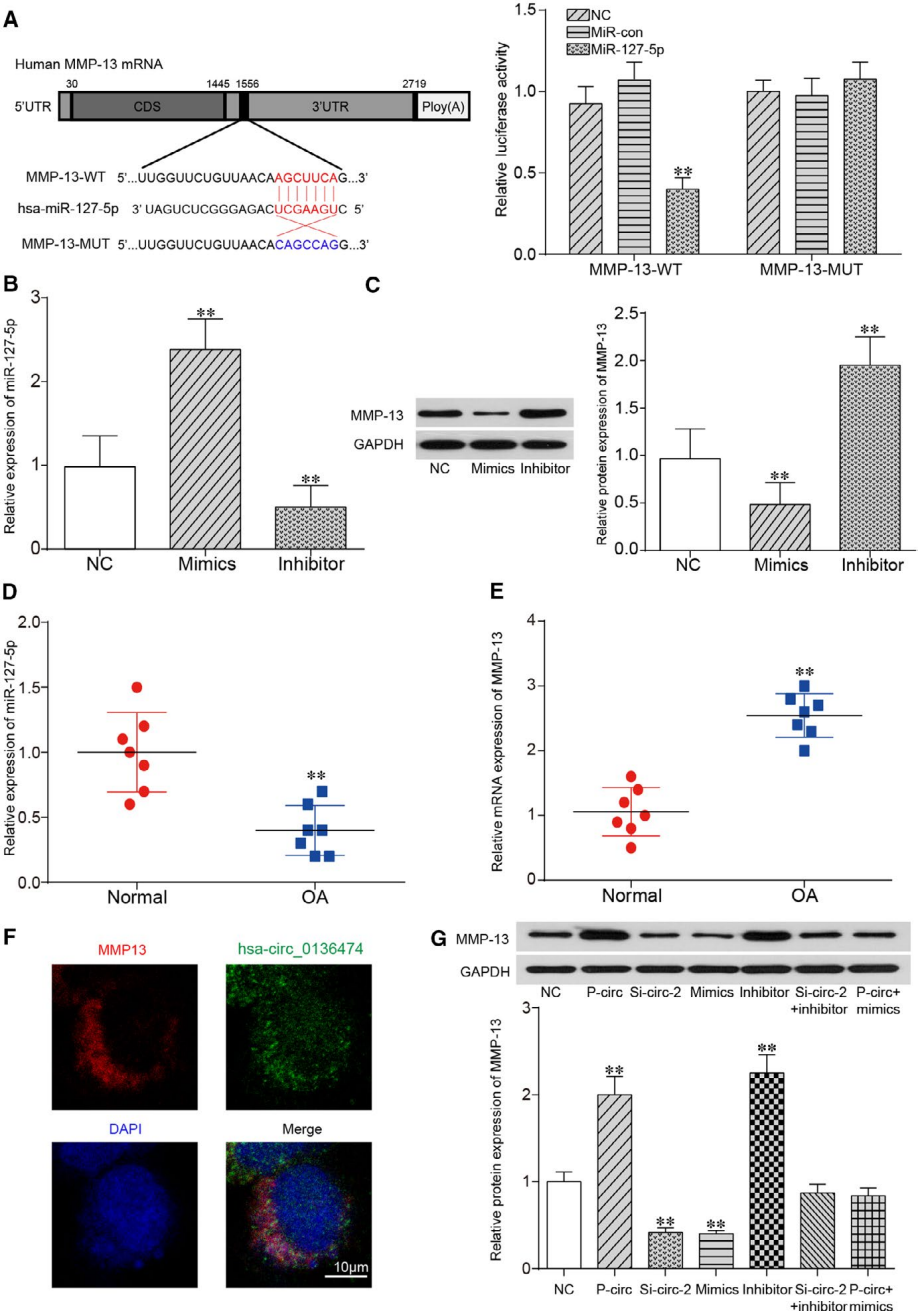
miR‐127‐5p could bind to MMP‐13 and suppress its expression. A, The targeted relationship between miR‐127‐5p and MMP‐13 using *Circular RNA Interactome* (https://circinteractome.nia.nih.gov/ ) and it was verified by dual‐luciferase reporter gene assay. B, The expression of miR‐127‐5p could be up‐regulated with miR‐127‐5p mimics yet down‐regulated with miR‐127‐5p inhibitor. C, Overexpression of miR‐127‐5p greatly down‐regulated the protein expression of MMP‐13, which could be instead up‐regulated with miR‐127‐5p inhibitor. D, E, Relative expression of miR‐127‐5p and MMP‐13 was tested in the normal cartilage tissues and OA cartilage tissues. F, RNA in situ hybridization reveals the immune‐localization of Circ_0136474 and MMP‐13 in OA tissues. Nuclei were stained with DAPI. Scale bar, 10 μm. G, The expression of MMP‐13 was tested by qRT‐PCR and Western blot. **P < 0.01 meant that there stood a significant difference compared with NC group

After analysing the sequence of MMP‐13, targeted relationship was uncovered between miR‐127‐5p seed region and MMP‐13 3′UTR region based on c*ircular RNA Interactome*. Relative luciferase activity in that group co‐transfected with miR‐127‐5p mimics and MMP‐13 WT was lower than that of group with co‐transfection of both miR‐127‐5p NC and MMP‐13 WT, suggesting the direct binding between miR‐127‐5p and MMP‐13 in OA (Figure 3A, *P* < 0.01). Thereafter, qRT‐PCR was adopted for validation of transfection efficiency. Overexpression of miR‐127‐5p tremendously increased miR‐127‐5p expression, which was largely reduced with inhibitor of miR‐127‐5p as shown in Figure 3B. Based on Figure 3C, inhibition of miR‐127‐5p greatly increased both mRNA expression and protein expression of MMP‐13, which was largely decreased with overexpression of miR‐127‐5p (*P* < 0.01). To summarize, miR‐127‐5p could directly target MMP‐13 and suppress its expression in OA. Thereafter, the relative expression of miR‐127‐5p and MMP‐13 in both OA cartilage tissues and normal cartilage tissues was measured as shown in Figure 3D,E respectively. miR‐127‐5p expression was significantly down‐regulated in OA cartilage tissues compared with in normal cartilage tissues (*P* < 0.01), while the expression of MMP‐13 was significantly higher in OA cartilage tissues than that in normal cartilage tissues (*P* < 0.01). We further performed RNA‐FISH in OA tissues to determine their immune‐localization. MMP‐13 was detected in the cytoplasm and Circ_0136474 was detected both in nucleus and cytoplasm. It showed an overlap in the expression of MMP‐13 and Circ_0136474, which indicated that the MMP‐13 and Circ_0136474 were co‐localized in the cytoplasm (Figure 3F). As shown in Figure 3G, overexpression of Circ_0136474 or inhibition of miR‐127‐5p greatly increased MMP‐13 protein expression, which was largely reduced with si‐Circ_0136474‐2 or miR‐127‐5p mimics (*P* < 0.01). In addition, si‐Circ_2 alone suppressed protein expression of MMP‐13, which could be rescued to normal expression level after co‐transfection with miR‐127‐5p inhibitor. Besides, p‐circ alone increased protein expression of MMP‐13, which could be retrieved to normal expression level after co‐transfection with miR‐127‐5p mimics. Taken together, Circ_0136474 sponged for up‐regulation of MMP‐13 in OA.

### 3.3 Overexpression of Circ_0136474 promoted cell apoptosis yet suppressed cell proliferation by promoting the expression of MMP‐13 yet suppressing miR‐127‐5p expression

**Figure 4:**
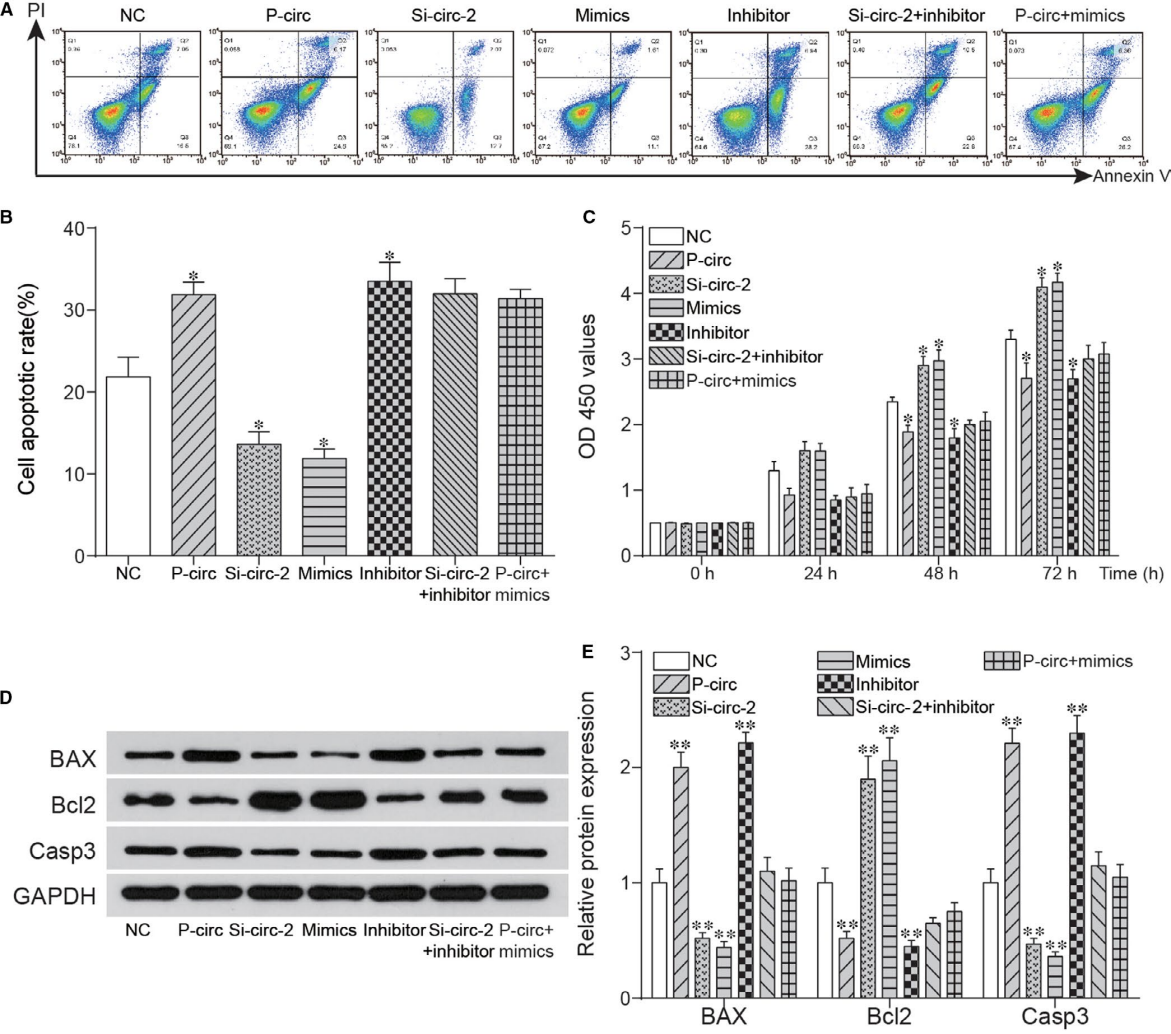
Circ_0136474 affected cell apoptosis and proliferation by down‐regulation of miR‐127‐5p. A, B, Si‐circ and miR‐127‐5p mimics reduced the cell apoptotic rate in OA cartilage tissues, while p‐circ and inhibitor enhanced the cell apoptotic rate markedly. C, The results of cell proliferation showed that si‐circ and miR‐127‐5p mimics could promote the cell proliferation and p‐circ or miR‐127‐5p inhibitor could suppress the cell proliferation while it was reverted by co‐transfection of si‐circ and inhibitor. D, E, The detection of expression level of apoptosis‐related proteins was included to ensure the effects on cell numbers in cell apoptosis were direct. *P < 0.05 and ** *P* < 0.01 showed there was a significant difference compared with NC group

**Figure 5:**
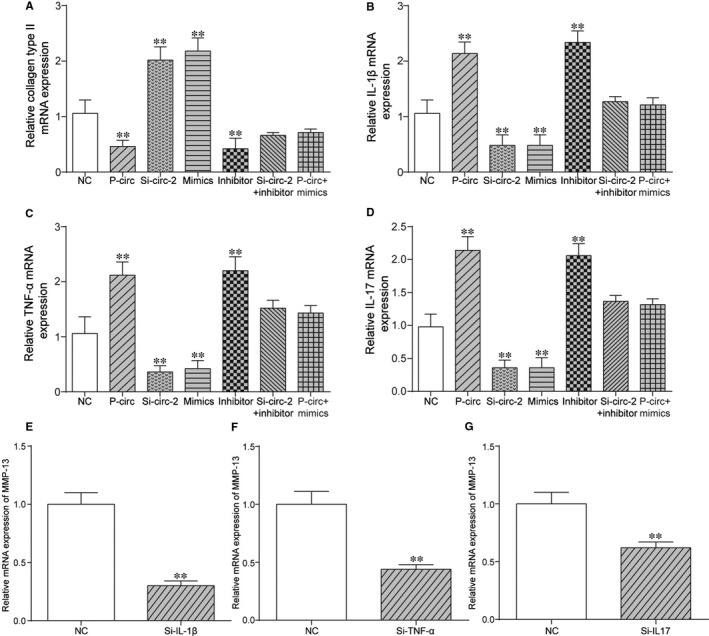
Circ_0136474 affected expression of inflammatory cytokines by down‐regulation of miR‐127‐5p. A‐D, Relative expression of collagen type II, IL‐1β, TNF‐α and IL‐17 was conducted via qRT‐PCR. (E‐G) Silencing TNF‐α, Il‐17 or IL‐1β could down‐regulate the expression of MMP‐13. **P < 0.01 showed there was a significant difference compared with NC group

To further investigate the roles of Circ_0136474/miR‐127‐5p in OA progression, flow cytometry and CCK‐8 assay were adopted for assessment of cell apoptotic rate and cell proliferation rate respectively. As shown in Figure 4A,B, either si‐Circ_0136474‐2 or miR‐127‐5p mimics could restrain cell apoptosis, which could be instead enhanced with the overexpression of Circ_0136474 or miR‐127‐5p inhibitor. Figure 4C reveals that the overexpression of Circ_0136474 or miR‐127‐5p inhibitor could suppress cell proliferation, which could be otherwise promoted with si‐Circ_0136474‐2 or miR‐127‐5p mimics. Besides, the relative expression levels of apoptosis‐related proteins, including Bax, Bcl2 and Caspase3 were further detected as shown in Figure 4D,E. The overexpression of Circ_0136474 or miR‐127‐5p inhibitor increased the relative protein expression of Caspase3 and Bax yet down‐regulated the expression of Bcl2, indicating the increase in cell apoptotic rate. In addition, silencing of Circ_0136474 or miR‐127‐5p mimics decreased cell apoptotic rate by down‐regulating protein expression of Caspase3 and Bax, while up‐regulating the expression of Bcl2. In addition, collagen type II, IL‐1β, TNF‐α and IL‐17 were inflammatory factors closely related with cell proliferation and cell apoptosis in OA. Based on Figure 5A, si‐Circ_0136474_2 or overexpression of miR‐127‐5p promoted the expression of Collagen type II, which was suppressed with overexpression of Circ_0136474 or inhibition of miR‐127‐5p (*P* < 0.01). Furthermore, si‐circ‐2 alone increased the expression of Collagen type II, which could be rescued to normal expression level after co‐transfection with miR‐127‐5p inhibitor. Besides, p‐circ alone decreased protein expression of MMP‐13, which could be recovered to normal expression level after co‐transfection with miR‐127‐5p mimics. Figure 5B‐D showed that si‐Circ_0136474‐2 or overexpression of miR‐127‐5p suppressed the expression of IL‐1β, TNF‐α and IL‐17, which was enhanced with overexpression of Circ_0136474 or inhibition of miR‐127‐5p (*P* < 0.01).

In addition, si‐Circ_2 alone suppressed expression of IL‐1β, TNF‐α and IL‐17, which could be retrieved to normal expression level after co‐transfection with miR‐127‐5p inhibitor. Besides, p‐circ alone up‐regulated the expression of IL‐1β, TNF‐α and IL‐17, which could be rescued to normal expression level after co‐transfection with miR‐127‐5p mimics. Furthermore, silencing of IL‐1β greatly down‐regulated the expression of MMP‐13 as shown in Figure 5E, indicating the potential positive feedback loop between IL‐1β and MMP‐13. As shown in Figure 5F,G, there might be potential positive feedback loop between IL‐17 and MMP‐13, as well as TNF‐α and MMP‐13. In short, overexpression of Circ_0136474 promoted cell apoptosis yet suppressed cell proliferation by promoting the expression of MMP‐13 yet suppressing miR‐127‐5p expression.

### 3.4 Silencing of MMP‐13 suppressed cell apoptosis yet enhanced cell proliferation via down‐regulation of IL‐1β, TNF‐α and IL‐17 and up‐regulation of Collagen type II

**Figure 6:**
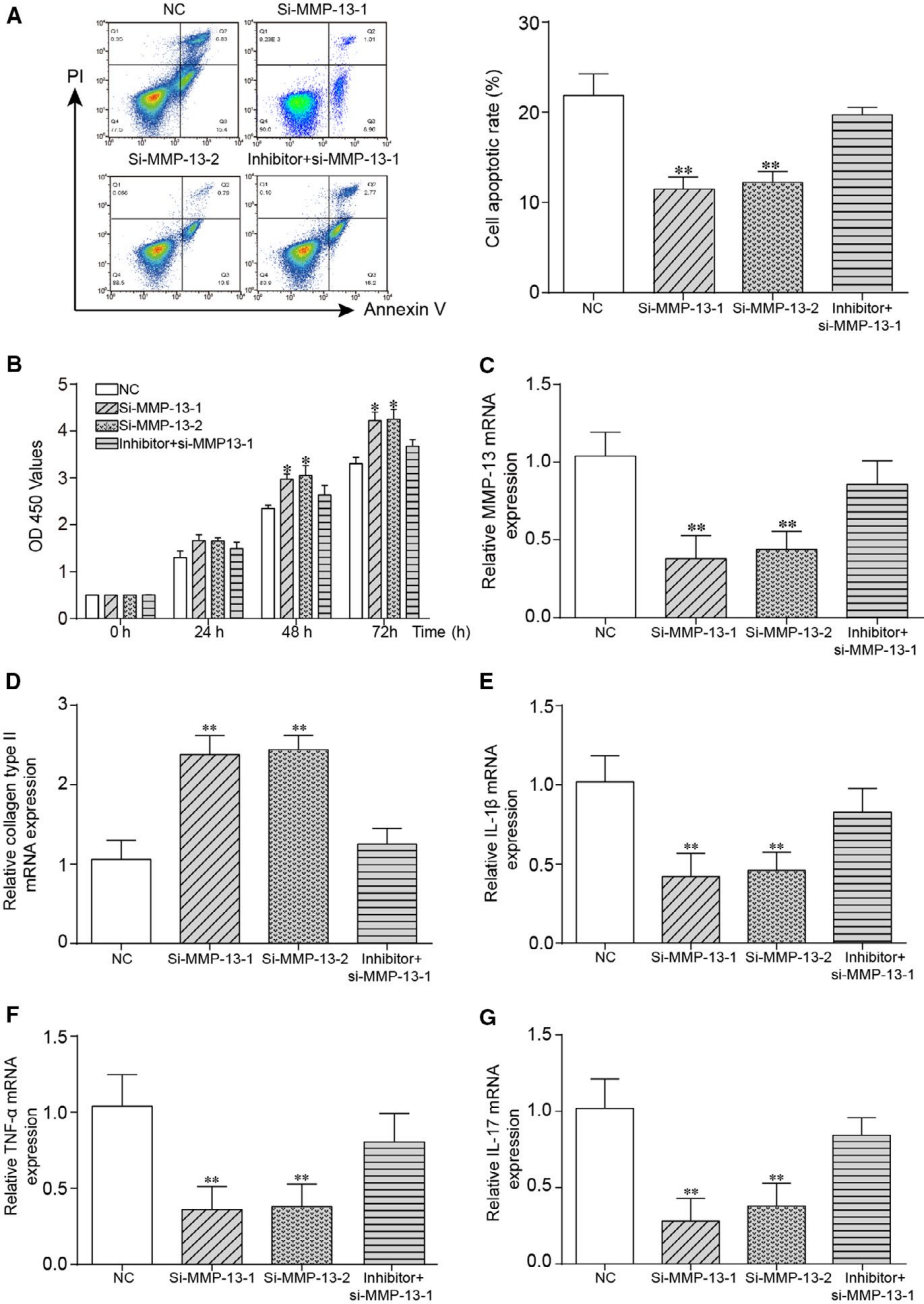
Circ_0136474 inhibited miR‐127‐5p to promote MMP‐13 expression in OA cartilage tissues. A, Si‐MMP‐13‐1 and si‐MMP‐13‐2 reduced cell apoptotic rate in OA cartilage tissues. B, The results of cell proliferation rate showed that si‐MMP‐13‐1 and si‐MMP‐13‐2 could promote the cell proliferation, while it was reverted by co‐transfection of inhibitor and si‐MMP‐13. C, It was verified that si‐MMP‐13‐1 and si‐MMP‐13‐2 could down‐regulate the expression of MMP‐13, which could be rescued to normal level with co‐transfection of inhibitor. D‐G, Relative expression of collagen type II, IL‐1β, TNF‐α and IL‐17 was performed via qRT‐PCR. *P < 0.05 and ** *P* < 0.01 showed that there was a significant difference compared with NC group

As shown in Figure 6A,B, si‐MMP‐13‐1 and si‐MMP‐13‐2 could up‐regulate cell proliferation rate yet decrease cell apoptotic rate, which could be reversed with co‐transfection with miR‐127‐5p inhibitor (*P* < 0.01). In addition, knockdown of MMP13 largely decreased MMP13 expression, which was retrieved to NC after co‐transfection with miR‐127‐5p inhibitor (*P* < 0.01) as shown in Figure 6C. Figure 6D showed that silencing MMP‐13 increased the expression of Collagen type II, which was rescued to NC after co‐transfection with inhibitor of miR‐127‐5p (*P* < 0.01). Based on Figure 6E‐G, down‐regulation of MMP‐13 reduced the expression of IL‐1β, TNF‐α and IL‐17, which was rescued to NC after co‐transfection with inhibitor of miR‐127‐5p (*P* < 0.01). In short, silencing MMP‐13 suppressed cell apoptosis yet enhanced cell proliferation via down‐regulation of IL‐1β, TNF‐α and IL‐17 and up‐regulation of Collagen type II.

## 4 DISCUSSION

It was unveiled by this study that both Circ_0136474 and MMP‐13 were significantly up‐regulated, whereas miR‐127‐5p expression was tremendously reduced in OA cartilage tissues compared with normal cartilage tissues. The overexpression of Circ_0136474 could suppress cell proliferation yet enhance cell apoptosis by inhibiting miR‐127‐5p expression and enhancing MMP‐13 expression in OA. Circ_0136474 sponged miR‐127‐5p for up‐regulation of MMP‐13 in OA. Knockdown of Circ_0136474 or silencing of MMP‐13 inhibited the expression of some inflammatory mediators, including IL‐1β, TNF‐α and IL‐17, while enhanced expression of collagen type II to suppress OA progression.

Circular RNAs (circRNAs), ubiquitously existing in the cytoplasm of eukaryotic cells are characterized by stable structure and high tissue‐specific expression. The aberrant expression of circRNAs was always accompanied with the development and progression of several types of diseases, such as atherosclerosis and nervous system disorders.^25^ Hence, circRNAs might well be selected as therapeutic targets, as well as new biomarkers. The circRNAs could regulate the expression of the mRNA by reducing the inhibitory effect of miRNAs.^26^ Our result revealed that Circ_0136474 was highly expressed in OA cartilage tissues compared with normal cartilage tissues and played its role in the cytoplasm. In addition, the overexpression of Circ_0136474 suppressed cell proliferation yet enhanced cell apoptosis by regulating inflammatory mediators and collagen type II in OA, which was similar to the stimulative roles of Circ_Atp9b in OA progression.^6^

miRNAs controlled a large set of biological processes through direct interaction with their target mRNAs. This regulation could occur by repressing the translation or by triggering the degradation of its target mRNAs. Previous studies showed that miRNAs played significant roles in the treatment of tumour.^19^ Our results showed that miR‐127‐5p was lowly expressed in OA cartilage tissues compared with normal cartilage tissues, indicating the suppressive role of miR‐127‐5p in OA progression. Targeting MALAT1 to rescue miR‐127‐5p expression in OA might inhibit chondrocyte proliferation through miR‐127‐5p‐mediated OPN regulation and downstream PI3K/AKT pathway.^27^ Based on latest research, miR‐27b was identified as a potential regulator of MMP‐13 expression in human chondrocytes by demonstrating its modulation by IL‐1β in human chondrocytes.^28^ Furthermore, it was reported that IL‐1β and TNF‐α were the two most potent catabolic factors that increased the production of inflammatory mediators and the expression of MMPs in chondrocytes.^29^ miR‐181b was a negative regulator of cartilage development and in vitro attenuation reduced MMP‐13 expression while inducing collagen type II expression.^30^ miR‐127‐5p promoted cell proliferation yet suppressed cell apoptosis by down‐regulation of MMP‐13 in OA, which was in line with results previously reported. In addition, knockdown of MMP‐13 inhibited the expression of some inflammatory mediators, including IL‐1β, TNF‐α and IL‐17, which meant that MMP‐13 could promote the expression of those inflammatory mediators above‐mentioned. Besides, silencing inflammatory mediators, such as IL‐1β, TNF‐α and IL‐17 could greatly down‐regulate MMP‐13 to affect OA progression, indicating that the positive feedback loop may exist between MMP‐13 and inflammatory mediators and it will provide us with new insight into regulatory relationships between inflammatory mediators and MMP‐13, as well as other matrix metalloproteinases in OA progression. In addition, the cascade of Circ_0136474/miR‐127‐5p/MMP‐13 in regulating cell proliferation and cell apoptosis in OA was illuminated preliminarily in our study.

However, there were also some limitations which should never be overlooked. The samples were not big enough for further verification of Circ_0136474/miR‐127‐5p/MMP‐13 in OA progression. Furthermore, the majority of experiments was carried out at the molecular level and cellular level, in vivo experiments were also required for further validation. In addition, it was not clear what really affected the expression alternation of Circ_0136474 and what the direct upstream or downstream targets of Circ_0136474/miR‐127‐5p/MMP‐13 might be in regulating the pathologies of OA. In addition, relative expression change in ASH2L mRNA and protein between OA cartilage tissues and normal cartilage tissues was not illuminated, which was not enough for the elucidation of the relationships between ASH2L and Circ_0136474 in OA. However, this part of experiment will be involved in further studies for further validation of roles of Circ_0136474/miR‐127‐5p/MMP‐13 in OA progression. Last, diagnosis and treatment of OA is far more sophisticated than our expectations. Therefore, it really does matter to identify new circRNAs, miRNAs and mRNAs, as well as interpret the mechanisms underlying. In short, Circ_0136474 could suppress cell proliferation yet enhance cell apoptosis by inhibiting miR‐127‐5p expression and enhancing MMP‐13 expression in OA.

## ETHICS APPROVAL AND CONSENT TO PARTICIPATE

This study was authorized by Peking University First Hospital and written informed consents were obtained from all the participants.

## CONFLICT OF INTEREST

No conflict of interest exits in the submission of this manuscript.

## Data Availability

The data that support the findings of this study are available from the corresponding author upon reasonable request.
