# Supplementary material for: Circ_0136474 and MMP‐13 suppressed cell proliferation by competitive binding to miR‐127‐5p in osteoarthritis
Source: J Cell Mol Med. 2019 Aug 11;23(10):6554–64. doi: 10.1111/jcmm.14400 (PMC6787461; doi:10.1111/jcmm.14400)
Supplement: Supplementary file 1 [file JCMM-23-6554-s001.docx]

**Supplementary Table 1. Predicted miRNAs targeting hsa_circ_0136474 using circular RNA Interactome.**

| hsa-miR-1228  hsa-miR-1231  hsa-miR-1238  hsa-miR-1248  hsa-miR-1264  hsa-miR-127-5p  hsa-miR-1281  hsa-miR-1288  hsa-miR-140-3p  hsa-miR-142-3p  hsa-miR-330-3p  hsa-miR-370  hsa-miR-384  hsa-miR-485-3p  hsa-miR-487a  hsa-miR-495  hsa-miR-513a-3p  hsa-miR-515-5p  hsa-miR-524-3p  hsa-miR-525-3p  hsa-miR-553  hsa-miR-567  hsa-miR-569  hsa-miR-57  hsa-miR-572  hsa-miR-582-3p  hsa-miR-593  hsa-miR-605  hsa-miR-607  hsa-miR-609  hsa-miR-618  hsa-miR-622  hsa-miR-623  hsa-miR-625  hsa-miR-626  hsa-miR-643  hsa-miR-647  hsa-miR-656  hsa-miR-659  hsa-miR-665  hsa-miR-668  hsa-miR-766  hsa-miR-873  hsa-miR-876-3p  hsa-miR-885-3p  hsa-miR-889  hsa-miR-942 |
| --- |
