# Supplementary material for: Circ_0136474 and MMP‐13 suppressed cell proliferation by competitive binding to miR‐127‐5p in osteoarthritis
Source: J Cell Mol Med. 2019 Aug 11;23(10):6554–64. doi: 10.1111/jcmm.14400 (PMC6787461; doi:10.1111/jcmm.14400)
Supplement: Supplementary file 2 [file JCMM-23-6554-s002.docx]

**Supplementary Table 2. Osteoarthritis-related miRNAs reported to date based on HMDD.**

| miRNAs | RefeRences |
| --- | --- |
| has-miR-146a | PMID: 28647559；PMID: 28785809 |
| has-miR-155 | PMID: 28647559；PMID: 28785809 |
| hsa-miR-140 | PMID: 22143896；PMID: 26723856 |
| hsa-miR-181a | PMID: 28785809 |
| hsa-miR-223 | PMID: 28785809 |
| hsa-miR-455 | PMID: 22143896 |
| hsa-miR-34a | PMID:27247228 |
| hsa-miR-210 | PMID:26244598 |
| hsa-miR-222 | PMID:26673737 |
| hsa-miR-23a | PMID:27318087 |
| hsa-miR-21 | PMID:25196583 |
| hsa-miR-122 | PMID:26239639 |
| hsa-miR-130a | PMID: 29532889 |
| has-miR-148a | PMID:24269634 |
| hsa-miR-188 | PMID:24928913 |
| hsa-miR-27a | PMID:19948051 |
| hsa-miR-27b | PMID:20131257 |
| hsa-miR-335 | PMID:26243143 |
| hsa-miR-483 | PMID:28139355 |
| hsa-miR-519b | [PMID:28423042](http://www.ncbi.nlm.nih.gov/pubmed/28423042) |
| hsa-miR-9 | PMID:27404795 |
| hsa-miR-125b | PMID:28260078 |
| hsa-miR-127 | PMID:24022470 |
| hsa-miR-145 | PMID:27922673 |
| hsa-miR-15a | PMID:27916780 |
| hsa-miR-16 | PMID:26350536 |
| hsa-miR-181 | PMID:28177757 |
| hsa-miR-199a | PMID:27515563 |
| hsa-miR-19a | PMID:27515563 |
| hsa-miR-204 | PMID:27999816 |
| hsa-miR-21 | PMID:24577233 |
| hsa-miR-24 | PMID:24572376 |
| hsa-miR-26a | PMID:26854724 |
| hsa-miR-30b | PMID:26653555 |
| hsa-miR-33a | PMID:25880168 |
| hsa-miR-370 | PMID:26103880 |
| hsa-miR-373 | PMID:26103880 |
| hsa-miR-92a | PMID:29241192 |
| hsa-miR-98 | PMID:27590063 |
| hsa-miR-101 | PMID:24018042 |
| hsa-miR-139 | PMID:26450708 |
| hsa-miR-335 | PMID:24582835 |
| hsa-miR-132 | PMID:20470394 |
| hsa-miR-15a | PMID:26707794 |
| hsa-miR-16 | PMID:20470394 |
| hsa-miR-183 | [PMID:23744481](http://www.ncbi.nlm.nih.gov/pubmed/23744481) |
| hsa-miR-223 | PMID:20470394 |
| hsa-miR-26b | [PMID:28000846](http://www.ncbi.nlm.nih.gov/pubmed/28000846) |
| hsa-miR-675 | [PMID:22527881](http://www.ncbi.nlm.nih.gov/pubmed/22527881) |
| hsa-miR-146b | [PMID:28085114](http://www.ncbi.nlm.nih.gov/pubmed/28085114) |
